# Molecular Characteristics and Pathogenicity of a Novel Recombinant Porcine Reproductive and Respiratory Syndrome Virus Strain from NADC30-, NADC34-, and JXA1-Like Strains That Emerged in China

**DOI:** 10.1128/spectrum.02667-22

**Published:** 2022-11-10

**Authors:** Jiankui Liu, Chen Liu, Ye Xu, Yuan Yang, Jiarui Li, Ailing Dai, Cuiqin Huang, Manlin Luo, Chunhua Wei

**Affiliations:** a College of Life Sciences, Longyan University, Longyan, Fujian, China; b Fujian Provincial Key Laboratory for the Prevention and Control of Animal Infectious Diseases and Biotechnology, Longyan University, Longyan, Fujian, China; c Engineering Research Center for the Prevention and Control of Animal Original Zoonosis, Fujian Province University, College of Life Science, Longyan University, Longyan, Fujian, China; d College of Animal Science, Fujian Agriculture and Forestry University, Fuzhou, Fujian, China; e College of Veterinary Medicine, South China Agricultural University, Guangzhou, Guangdong, China; Oklahoma State University, College of Veterinary Medicine

**Keywords:** cross-protection, NADC30-like, NADC34-like, pathogenicity, porcine reproductive and respiratory syndrome virus, viral genome recombination

## Abstract

Recently, the emergence of a NADC34-like porcine reproductive and respiratory syndrome virus (PRRSV), which causes a large number of abortions in swine herds, has raised great concern in China. In this study, a PRRSV variant strain, PRRSV/CN/FJGD01/2021, evolved from recombination between NADC30-like, NADC34-like, and JXA1-like viruses was isolated in Fujian province in 2021, and its pathogenicity in piglets was examined. Animal experiments demonstrated that PRRSV/CN/FJGD01/2021 infection could induce 100% morbidity and cause higher viremia, a persistently higher fever (>40°C for 14 consecutive days), significant weight loss, and severe histopathological lung lesions compared to the NADC30-like FJZ03 strain and NADC34-like FJ0908 strain in piglets. The PRRSV/CN/FJGD01/2021 strain displayed higher pathogenicity than the FJZ03 and FJ0908 strains, but lower pathogenicity than the Chinese highly pathogenic (HP)-PRRSVs in piglets. Moreover, the Ingelvac PRRS modified live vaccine (MLV) provides incomplete cross-protection against heterologous PRRSV/CN/FJGD01/2021 in piglets. Our findings contribute to the understanding of the current epidemic situation of NADC34-like PRRSV in China.

**IMPORTANCE** The pathogenicity of NADC34-like PRRSV has broad variations in virulence. Importantly, NADC34-like PRRSV has undergone complex recombination with local strains since it first emerged in 2017 in China. However, the pathogenicity of the recombinant NADC34-like virus was rarely experimentally evaluated in pigs. In this study, a novel PRRSV strain, PRRSV/CN/FJGD01/2021, was isolated from sows enduring a high-abortion-rate (20%) period in China in 2021. Notably, phylogenetic and recombination analyses revealed that PRRSV/CN/FJGD01/2021 is a recombinant virus from NADC30-, NADC34-, and JXA1-like isolates. PRRSV/CN/FJGD01/2021 was shown to cause higher virus load, persistent fever, significant weight loss, moderate respiratory clinical signs, and severe histopathological lung lesions in piglets. PRRSV/CN/FJGD01/2021 exhibited higher pathogenicity than NADC30-like FJZ03 and NADC34-like FJ0908, but lower than Chinese HP-PRRSVs for piglets. These data indicated that PRRSV/CN/FJGD01/2021 has intermediate virulence for piglets. Furthermore, the Ingelvac PRRS MLV could partly provide protective efficacy against PRRSV/CN/FJGD01/2021 challenge in piglets.

## INTRODUCTION

Porcine reproductive and respiratory syndrome (PRRS) is generally considered one of the most important infectious diseases in pigs, as it causes severe economic losses worldwide each year. PRRS virus (PRRSV), the causative agent of PRRS, was first identified in Europe and the United States in the early 1990s (1, 2). PRRSV is an enveloped, single-stranded, positive-sense RNA virus belonging to the *Arteriviridae* family (3, 4).

The size of the PRRSV genome is 15.0 to 15.5 kb with a 5′ and a 3′ untranslated region. The PRRSV genome comprises at least ten overlapping open reading frames (ORFs) including ORF1a, ORF1b, ORF2a, ORF2b, ORF3, ORF4, ORF5a, and ORF5-ORF7 (4–6). Among these ORFs, the two large ORFs, ORF1a and ORF1b, occupy approximately 75% of the genome and encode at least 16 nonstructural proteins (NSPs), including Nsp1α, Nsp1β, Nsp2, Nsp2TF, Nsp2N, Nsp3 to 6, Nsp7α, Nsp7β, and Nsp8 to 12 (7, 8). Other ORFs are located at the 3′ terminus and encode eight structural proteins: GP2, E, GP3, GP4, GP5a, GP5, M, and N (5, 7, 9, 10).

Generally, PRRSVs are classified into two species: *Betaarterivirus suid 1* (PRRSV-1, European virus) and *Betaarterivirus suid 2* (PRRSV-2, North American virus), with the latter being predominant in China since its initial recognition there in 1996 (11, 12). According to the global PRRSV classification system, the Chinese PRRSV-2 strains can be classified into lineage 1 (NADC30-like), lineage 3 (QYYZ-like), lineage 5 (VR2332-like), and lineage 8 (JXA1-like and CH-1a-like) (13, 14). Highly pathogenic PRRSV (HP-PRRSV/JXA1-like/sublineage 8.7), characterized by high fever and morbidity, emerged in China in 2006, causing enormous economic losses in swine herds (15, 16). In 2012, the NADC30-like PRRSV (sublineage 1.8) was first reported in China; it spread rapidly throughout China and is currently the most important PRRSV in swine herds (13, 17–19). Recently, PRRSV isolates from ORF5 RFLP 1-7-4 (NADC34-like) viruses belonging to sublineage 1.5, which emerged in the United States, causing dramatic abortion “storms” in sow herds (20, 21). NADC34-like PRRSV was first reported in China in 2017 and subsequently spread rapidly to multiple provinces in China (22–24). To date, sublineage 8.7 (JXA1-like) and sublineage 1.8 (NADC30-like) have become the dominant strains circulating in China’s swine herds, and NADC30-like strains have received great attention in recent years because of their high rate of recombination and mutation. In recent years, diverse novel recombinant PRRSV strains originating from NADC30-like PRRSV outbreaks in China have been reported and have demonstrated different pathogenicity (25–30).

In July 2021, we isolated a novel PRRSV strain, PRRSV/CN/FJGD01/2021, from a pig farm experiencing a large abortion outbreak (an abortion rate of approximately 20%) in Fujian Province, China. This pig farm was vaccinated three times per year with the Ingelvac PRRS modified live vaccine (MLV) to control PRRS. Phylogenetic and molecular evolutionary analyses indicated that PRRSV/CN/FJGD01/2021 is a natural recombinant event among NADC30-, NADC34-, and JXA1-like strains. To further understand the PRRSV/CN/FJGD01/2021 strain, we genetically characterized the complete genome of the virus and explored its pathogenicity in piglets. Additionally, a commercial PRRSV modified live vaccine (Ingelvac PRRS MLV) that has been widely used in China was used to evaluate the potential of cross-protective efficacy against PRRSV/CN/FJGD01/2021 under experimental conditions.

## RESULTS

### Genomic characteristics of PRRSV/CN/FJGD01/2021.

The full-length genomic sequence of PRRSV/CN/FJGD01/2021 (GenBank accession number OL310959) was 15,019 nucleotides (nt), excluding the poly(A) tails at the 3′ end. Comparative sequence analyses showed that PRRSV/CN/FJGD01/2021 had an identity of 84.7% with VR-2332, 84.6% with JXA1, 84.6% with CH-1a, 90.5% with NADC30, 86.3% with NADC34, 82.1% with QYYZ, and only 60.6% with the Lelystad virus (LV) (PRRSV-1 representative strain) (Table 1). Notably, comparison to PRRSV sequences in the GenBank database revealed that the PRRSV/CN/FJGD01/2021 strain grouped in the NADC30-like PRRSVs.

**TABLE 1:** Sequence distance of the PRRSV/CN/FJGD01/2021 isolate with seven PRRSV reference strains^*a*^

Genome region^*b*^	Pairwise identity to PRRSV-CN-FJGD01-2021 (%)													
	VR2332		JXA1		CH-1a		NADC30		NADC34		QYYZ		LV	
	nt identity	aa identity	nt identity	aa identity	nt identity	aa identity	nt identity	aa identity	nt identity	aa identity	nt identity	aa identity	nt identity	aa identity
Complete genome	84.9		84.6		84.6		***90.5***		86.3		82.1		60.6	
5′ UTR	93.1		92.1		***93.2***		98.9		95.7		90		62.4	
ORF1a	82.3	82.5	82.3	82.4	82.1	82.1	***88.6***	***89.9***	81.0	81.8	78.6	79.4	55.4	49.1
ORF1b	86.5	94.9	86.9	95.7	87.3	95.1	***93.9***	***97.8***	88.0	95.4	85.1	94.2	64.1	68.8
ORF2a	87.5	85.9	85.6	82.8	85.9	85.2	84.7	82.8	***95.2***	***93.0***	85.3	83.6	66.0	62.7
ORF2b	90.1	87.8	86.9	83.8	86.5	85.1	86.5	89.2	***96.8***	***97.3***	87.8	89.2	71.4	73.2
ORF3	83.4	79.9	83.1	79.1	83.0	79.1	84.6	83.9	***94.2***	***92.1***	82.2	81.5	64.8	55.7
ORF4	86.8	87.6	85.5	87.1	86.0	87.1	95.0	95.5	***96.1***	***96.6***	84.7	85.4	67.2	69.7
ORF5	86.6	84.0	85.7	87.0	86.2	86.0	86.9	89.0	***95.9***	***93.0***	84.6	84.5	63.1	56.1
ORF5a	88.5	86.3	87.2	80.4	88.7	87.0	88.7	91.3	***94.3***	***95.7***	89.1	88.2	59.8	48.8
ORF6	89.7	94.8	88.4	94.8	87.2	93.1	94.9	96.0	***94.9***	***97.1***	89.7	94.8	70.9	80.3
ORF7	91.1	92.7	90.1	91.1	90.1	92.7	***96.8***	***95.9***	95.2	93.5	87.4	90.2	67.3	63.3
3′ UTR	92.7		90.0		88.1		***96.7***		94.0		88.1		72.6	

a Each segment sequence of the reference strains sharing the highest similarity to PRRSV/CN/FJGD01/2021 is shown in bold italic.

b UTR, untranscribed region.

**FIG 1:**
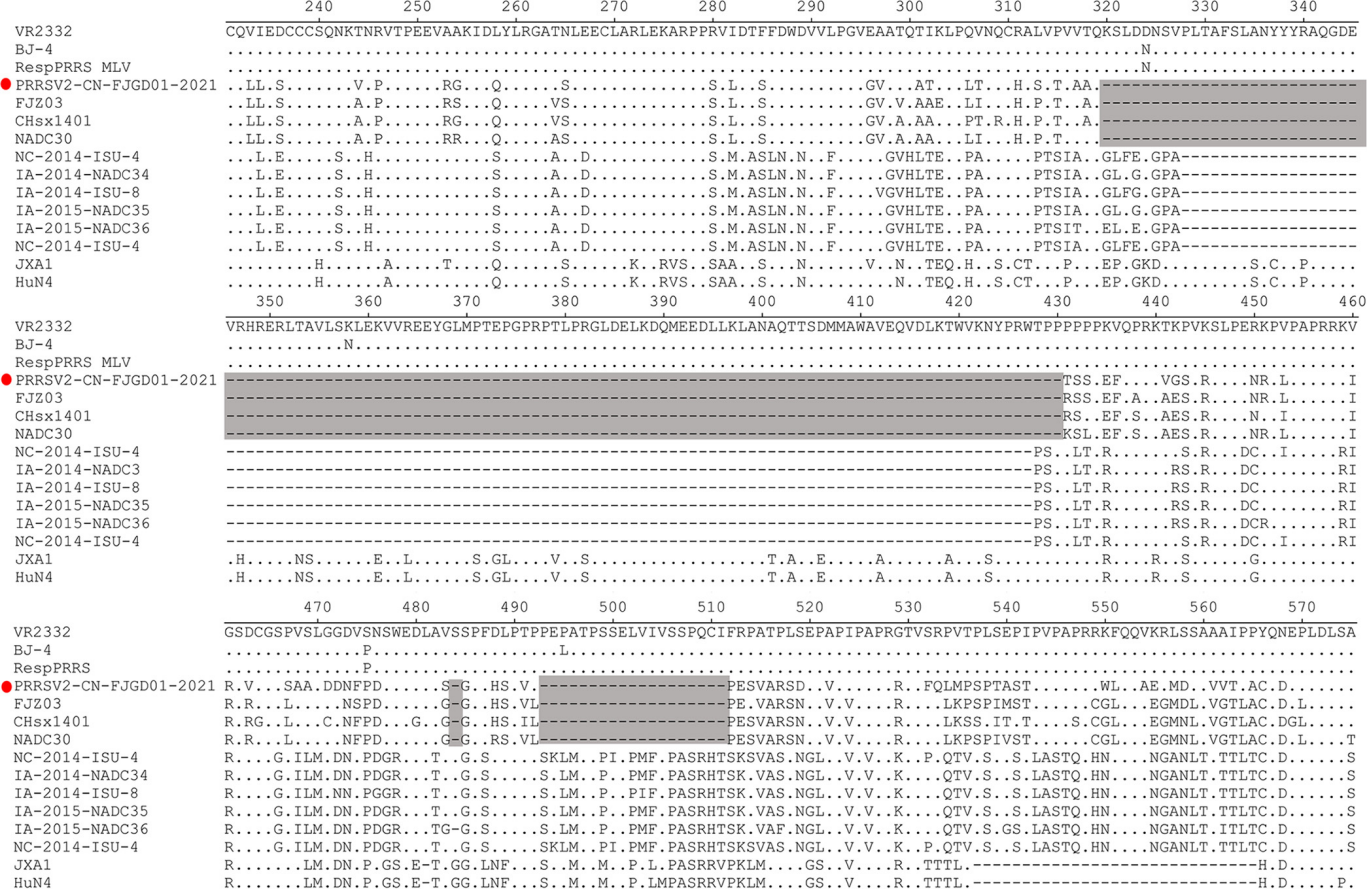
PRRSV/CN/FJGD01/2021 and NADC30-like PRRSVs had three discontinuous 131-aa deletion patterns in the Nsp2 gene. The 131-aa deletion sites are marked with gray rectangles. The PRRSV/CN/FJGD01/2021 strain in this study is labeled with red circle.

To better understand the genomic characteristics of PRRSV/CN/FJGD01/2021, the viral genome was analyzed in detail compared to the VR-2332, CH-1a, JXA1, NADC30, NADC34, and LV viruses (Table 1). The sequences from the different parts of the PRRSV/CN/FJGD01/2021 genome showed various percentages of identity to different sublineages. ORF1a, ORF1b, and ORF7 of PRRSV/CN/FJGD01/2021 shared a high identity percentage compared to NADC30; in contrast, ORFs 2 to 6 were closer to the NADC34 strain than to the representative PRRSV-2 sequences (Table 1). Strikingly, the Nsp2 protein of strain PRRSV/CN/FJGD01/2021 contained a discontinuous 131-amino acid (aa) deletion compared to the reference strain VR-2332, a unique characteristic of NADC30 and NADC30-like strains (Fig. 1).

### Viral growth kinetics.

The growth properties of PRRSV/CN/FJGD01/2021, FJZ03, and FJ0908 were examined on porcine alveolar macrophages (PAMs) and MARC-145^CD163^ cells. PRRSV/CN/FJGD01/2021 exhibited a faster and higher replication rate than FJZ03 and FJ0908 from 36 to 72 h postinfection in PAMs and MARC-145^CD163^ cells (data not shown).

### Recombination analysis.

**FIG 2:**
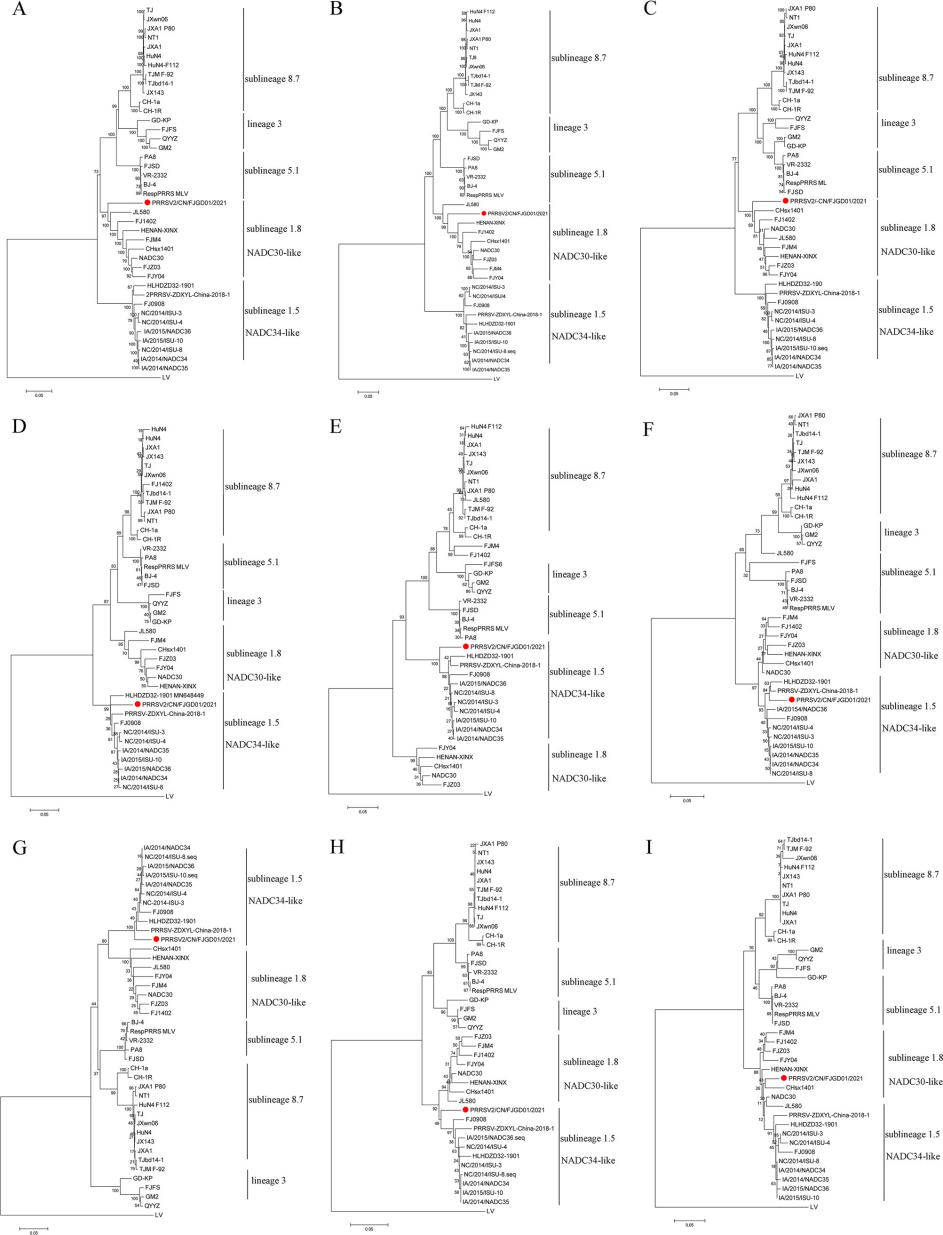
Phylogenetic analyses of PRRSV/CN/FJGD01/2021. (A to I) Phylogenetic trees were constructed separately based on the complete genome (A), ORF1a (B), ORF1b (C), ORF2 (D), ORF3 (E), ORF4 (F), ORF5 (G), ORF6 (H), and ORF7 (I). The reliability of the tree was assessed by bootstrap analysis of 1,000 replications. The PRRSV/CN/FJGD01/2021 strain in this study is labeled with a red circle.

Phylogenetic trees were constructed based on the whole genome and eight genomic regions using the sequences of 36 representative PRRSV isolates. Whole-genome phylogenetic analysis indicated that PRRSV/CN/FJGD01/2021 clustered as PRRSV-2 sublineage 1.8 (Fig. 2). PRRSV/CN/FJGD01/2021 grouped in sublineage 1.8 with the representative strain NADC30 in the ORF1a, ORF1b, and ORF7 regions, whereas it clustered in sublineage 1.5 with the representative strain NADC34 in the ORF2, ORF3, ORF4, ORF5, and ORF6 regions (Fig. 2). These findings suggest that the PRRSV/CN/FJGD01/2021 strain is a mosaic strain.

**FIG 3:**
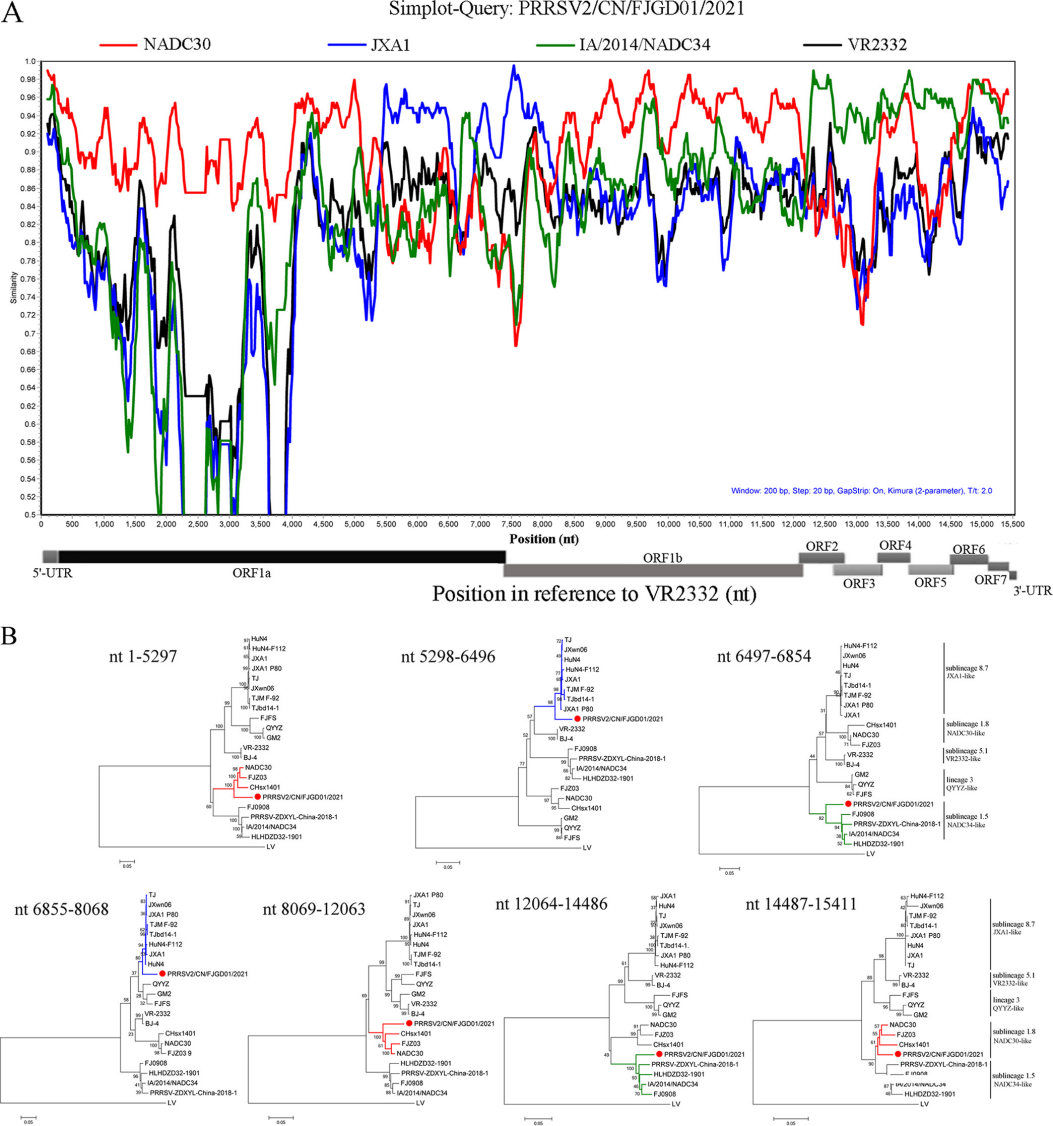
Genome recombination analysis of PRRSV/CN/FJGD01/2021. (A) Comparison of genetic similarity between PRRSV/CN/FJGD01/2021 and parental strains using SimPlot. For similarity plot analysis, PRRSV/CN/FJGD01/2021 was chosen as a query sequence, and the *y* axis indicates the percentage identity in a sliding 200-bp window with 20-bp steps. (B) Phylogenetic trees based on different regions of PRRSV/CN/FJGD01/2021. The PRRSV/CN/FJGD01/2021 strain in this study is labeled with a red circle.

The recombination detection program (RDP 4.10) and SimPlot 3.5.1 software were used to confirm the possible recombination events within the PRRSV/CN/FJGD01/2021 strain. A recombination analysis revealed six potential recombination breakpoints at nucleotide positions (with reference to the VR-2332 strain) 5297 (Nsp3), 6496 (Nsp5), 6854 (Nsp7), 8068 (Nsp9), 12063 (Nsp12), and 14486 (ORF6) (Table 2). Additionally, these recombination events were supported by similarity plots in SimPlot 3.5.1 and statistically incongruent phylogenetic trees (Fig. 3A and B). The six breakpoints separated the genome of PRRSV/CN/FJGD01/2021 into seven regions. The three regions between the breakpoints (nt 1 to 5297, nt 8069 to 12063, and nt 14487 to 15411) grouped in the NADC30-like strains cluster, and two regions (nt 5298 to 6496 and nt 6855 to 8068) clustered with JXA1-like strains, but the last two regions between the breakpoints (nt 6497 to 6854 and nt 12064 to 14486) belonged to NADC34-like strains with reference to the VR-2332 strain (Fig. 3B).

**TABLE 2:** Information on recombination events of isolate PRRSV/CN/FJGD01/2021

Breakpoint position in alignment		Major parent	Minor parent	*P* value						
Beginning	Ending			RDP	GENECONV	Bootscan	MaxChi	Chimaera	SiScan	3Seq
5297	6496	NADC30	JXA1	2.079 × 10^−51^	1.551 × 10^−31^	6.707 × 10^−48^	3.354 × 10^−18^	2.717 × 10^−10^	2.804 × 10^−23^	9.059 × 10^−14^
6854	8068	NADC30	JXA1	2.130 × 10^−60^	1.926 × 10^−34^	1.308 × 10^−61^	4.279 × 10^−17^	6.296 × 10^−3^	2.525 × 10^−21^	9.059 × 10^−14^
12063	14486	NADC30	NADC34	1.607 × 10^−61^	3.297 × 10^−17^	7.380 × 10^−46^	1.279 × 10^−24^	4.563 × 10^−26^	5.780 × 10^−19^	3.623 × 10^−13^

### Clinical signs.

**FIG 4:**
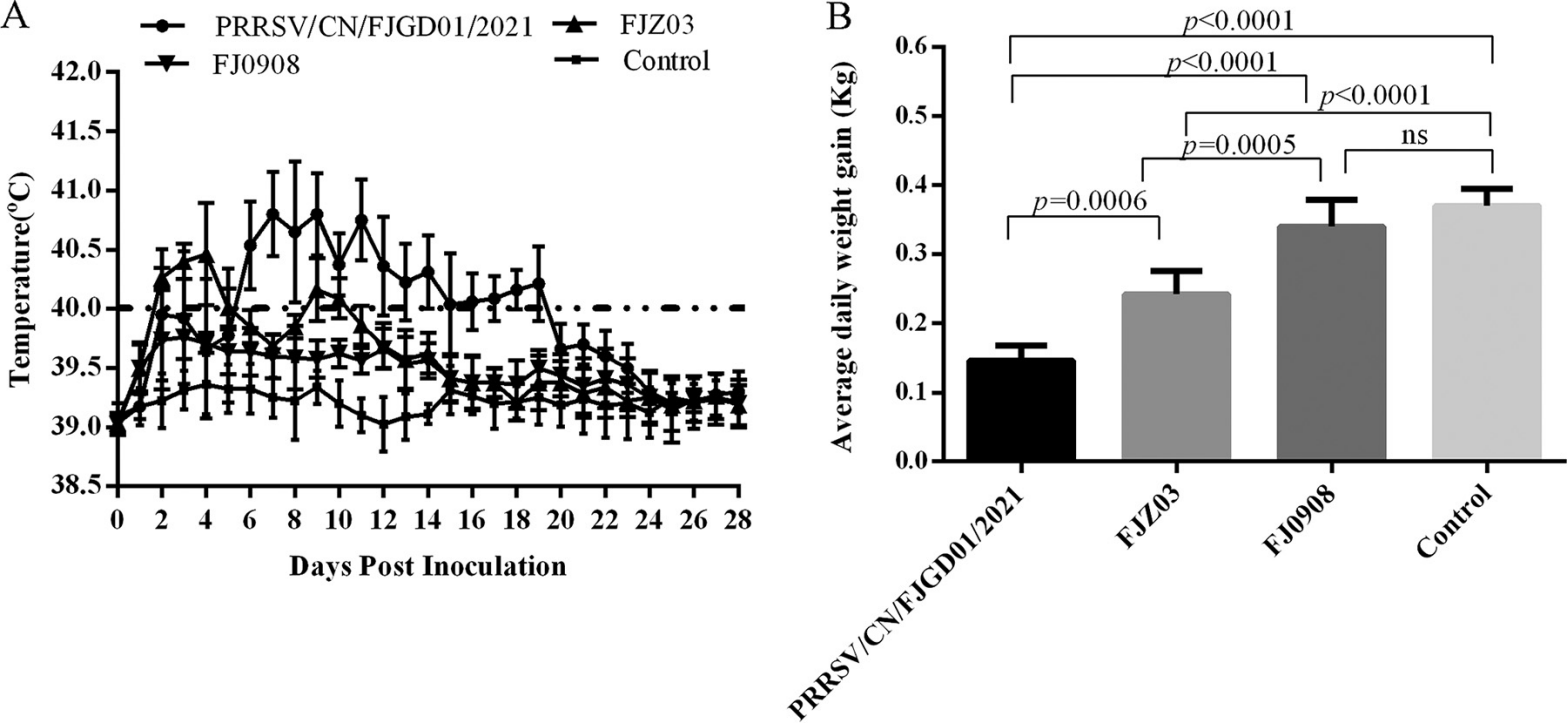
Rectal temperature and weight gain of piglets in different groups during the challenge study. (A) Mean rectal temperature after challenge with PRRSV/CN/FJGD01/2021, FJZ03, and FJ0908 viruses compared with uninfected controls. Data are expressed as the mean ± SD (error bars) of every group, and the fever cutoff value was set at 40.0°C. (B) Average daily weight gain of the experimental groups in the study. Data are expressed as the mean ± SD (error bars) of every group.

Piglets infected with PRRSV/CN/FJGD01/2021 exhibited obvious clinical signs starting at 2 days postinoculation (dpi), with a persistent high fever (>40.0°C, ranging from 40.1°C to 41.4°C) between 6 and 19 dpi, coughing, lethargy, dyspepsia, and shivering in all infected piglets. The average rectal temperature of piglets infected with PRRSV/CN/FJGD01/2021 was significantly higher than that of the FJZ03-infected, FJ0908-infected, and control piglets between 6 and 19 dpi (*P* < 0.05) (Fig. 4A). However, piglets inoculated with FJZ03 showed moderate clinical signs, including coughing, lethargy, and mild anorexia between 3 and 7 dpi. Compared with the FJ0908 group, the piglets inoculated with FJZ03 exhibited significantly elevated temperatures at 2, 3, 4, 5, 9, 10, and 11 dpi (*P* < 0.05) (Fig. 4A). In contrast, the average body temperature of piglets in the FJ0908-infected and control groups remained within the range of 39.0 to 39.7°C, and no obvious clinical signs were observed during the study. All piglets in this study survived until the end of the experiment.

Piglets infected with PRRSV/CN/FJGD01/2021, FJZ03, and FJ0908 strains displayed average daily weight gains of 0.146, 0.242, and 0.340 kg, respectively (Fig. 4B). As shown in Fig. 4B, the control piglets showed an average daily gain of 0.370 kg. Compared with the control group, the FJ0908-infected group showed only a slight decrease in weight gain, whereas the PRRSV/CN/FJGD01/2021- and FJZ03-infected groups showed a significant decrease in weight gain (*P* < 0.01). Furthermore, PRRSV/CN/FJGD01/2021-infected piglets had significantly lower weight gain than the FJZ03-infected piglets (*P* < 0.01) (Fig. 4B).

### Viral loads in serum samples, nasal secretions, and lung samples.

**FIG 5:**
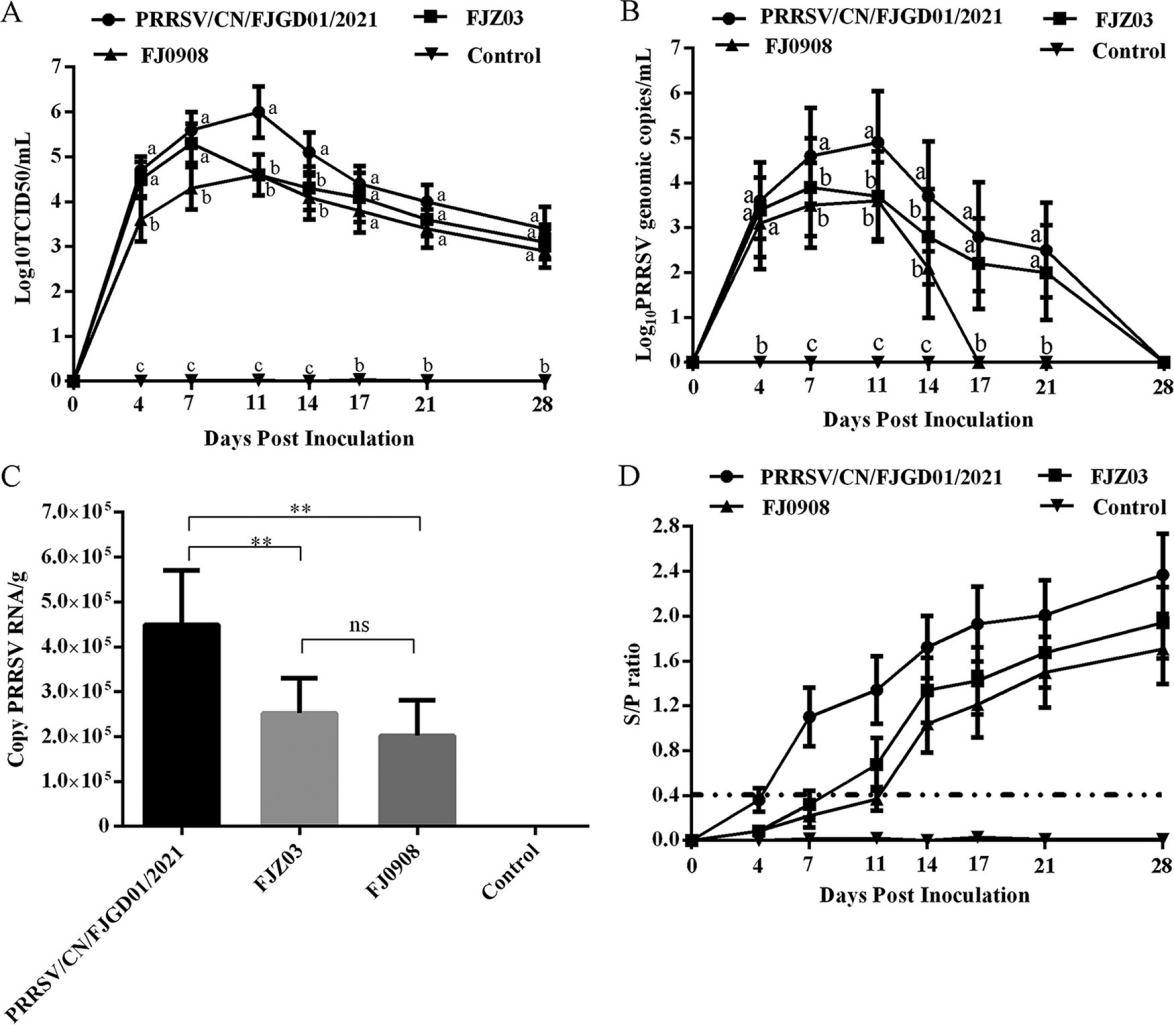
Dynamics of viral loads and antibody level of the infected piglets during the course of the study. (A) Evolution of mean viral load in serum after challenge. Serum samples were collected prior to infection and at 0, 4, 7, 11, 14, 17, 21, and 28 dpi. The virus loads are expressed as 50% of the tissue culture infective dose per mL (TCID_50_/mL) of sample through IFA. Different letters (a, b, c, d) indicate that groups are significantly different from each other, with a *P* value of <0.05. (B) Evolution of the mean genomic copy number of PRRSV RNA in inoculated piglets from PRRSV/CN/FJGD01/2021, FJZ03, FJ0908, and control groups in nasal swab (relative amount expressed in log_10_ R) after inoculation (day 0). Nasal swab samples were collected prior to infection and at 0, 4, 7, 11, 14, 17, 21, and 28 dpi. Different letters (a, b, c, d) indicate that groups are significantly different from each other, with a *P* value of <0.05. (C) The mean genomic copy number of PRRSV RNA in inoculated piglets from PRRSV/CN/FJGD01/2021, FJZ03, FJ0908 and control groups in lungs at 28 dpi. The significant difference is marked with the asterisk (*, *P < *0.05; **, *P < *0.01); (D) PRRSV-specific N protein antibody level after challenge. Serum samples were collected prior to infection and at 0, 4, 7, 11, 14, 17, 21, and 28 dpi. The threshold for seroconversion was set at S/P ratio of 0.4. Data are expressed as the Mean ± SD (error bars) of every group.

Blood samples and nasal secretions were collected at 0, 4, 7, 11, 14, 17, 21, and 28 dpi in every PRRSV-infected group to detect viremia in piglets. PRRSV was isolated from the serum of all piglets infected with PRRSV/CN/FJGD01/2021, FJZ03, and FJ0908 at 4 dpi, and mean virus titers reached peak levels at 11 dpi (10^5.9^ 50% tissue culture infective dose [TCID_50_]/mL), 7 dpi (10^5.3^ TCID_50_/mL), and 11 dpi (10^4.6^ TCID_50_/mL), respectively; the viremia persisted until 28 dpi in every PRRSV-infected group (Fig. 5A). Furthermore, the mean virus loads for PRRSV/CN/FJGD01/2021 piglets were significantly higher than those of the FJZ03- and FJ0908-inoculated groups at 11 and 14 dpi (*P* < 0.05). Meanwhile, no PRRSV was detected in the control group in serum throughout the study.

To investigate virus shedding in different groups, the viral RNA in nasal swab samples was quantified by quantitative PCR (qPCR). Viral RNA was detected in all piglets in the PRRSV/CN/FJGD01/2021- and FJZ03-infected groups between 4 and 21 dpi and reached the highest levels at 11 dpi and 7 dpi, respectively. At 4 dpi, only two piglets in the FJ0908-infected group had virus-positive nasal swab samples; all piglets had virus-positive nasal swab samples at 7, 11, and 14 dpi, three piglets had positive samples at 17 dpi, and none were observed to have positive nasal samples at 21 and 28 dpi. There was a significant difference in the virus detection levels between the PRRSV/CN/FJGD01/2021-infected and FJZ03- and FJ0908-infected groups at 7, 11, and 14 dpi (*P* < 0.05) (Fig. 5B).

Viral load was also detected in the lungs of all infected pigs in each group. The mean viral load in the lungs of piglets in the PRRSV/CN/FJGD01/2021-infected group was significantly higher than that in the FJZ03- and FJ0908-infected groups (*P* < 0.05; Fig. 5C). In contrast, viral RNA was not detected in the lung tissue samples of the control group. Additionally, PRRSVs were isolated from the serum and lung samples of piglets challenged with three strains, and ORF5 and ORF7 (Table 3) genes were amplified from virus, serum, and lung samples and sequenced to confirm that they were the inoculated original virus used in the study.

**TABLE 3:** List of primers used in this study

Fragment	Sequence (5′–3′)	Location^*a*^	Length of PCR products (bp)
A	GAATTCATGACGTATAGGTGTTGGCTAGCATGTCCACCCTATCCCAC	1–3021	3,021
B	CGTCAAGCAGCTCTCTGTCA5′- GACGAGGTTTGAGGTAGAATAGC	2811–4465	1,655
C	TGGCCAAAGCCCTATTGAG5′- AAGGGCTGCCGTCAGAAGCCTGA	4150–6057	1,908
D	TTCCTCCTGTGGAGAATGATGG CCTCACCGCTGTCTCAGTAACAACT	5873–7372	1,500
E	CAGGGCCTGACTAAGGAACAG CTGCAAGAATCCTGTCMCGG	7274–9010	1,737
F	TTCAGACAGACCCAAAGAAGACGCCAAGTTGACAGGCGTGCT	8902–10402	1,501
G	ATTCAACCCGATTACAGAGACAAGC CTGAGCAGTTACAGAAGATCTTATG	10084–11906	1,822
H	CCATCTTGTTTGGCTTCAC CAGCTCACATATCGTCAGGTT	11786–13545	1,760
I	GGTATGTTGGGGAAATGCTTGACCG TTAATTACGGCCGCATGGTTCTC	13393–15019	1,627
J	ATGACGTATAGGTGTTGGCTCTATGC AGCTTTCTCAAGCCTAGCCAAGCATT	1–2167	2,167
K	GGGCAACTGATGAAGATCTTG AATCGATGAGTGATGACCTGG	1593–4076	2,484
L	CAAGCAGCTCTCTGTCAAGC CTCACCGCTGTCTCAGTAACAACT	2814–7370	4,557
M	CGCCCCTCAGGCCAGTTCTGTAA GCTAGGGGTCTTATAAGGTATGTC	5660–7815	2,156
N	GTTGAGCTAAAAGACGCGATTGA GTGGTGCCAAAACATTCCTAAGG	7435–11983	4,549
O	AGGACTGGGAGGATTACAAT CGGACGACAAATGCGTGGTTAT	11549–14390	2,842
P	GATAACCACGCATTTGTCGT TATAGCGGCCGCTTTTTAATTWCGGCC	14367–15019	653
Break point 596	CCCAACACGGTCTTACACTACC GTCAATAGTGAACACTCCGCCA	4716–5311	596
Break point 625	CCGCTCGGTGATGTGAAAATC CCTTCCCTCAACTTCCCTTCA	5738–6363	625
Break point 321	TGCGATACTTTGCTGAAGGGA CAATGTCACCGGGCGAGATT	6330–6650	321
Break point 706	ACTGACTGCCAAAGAACTGGA GCACATACAACTCAAACCCGG	7225–7930	706
Break point 520	GTCTAGTGCATACCATGGTGA AGGGGAAGGAAAACCTCATAA	11403–11922	520
Break point 775	CTGCGTTAACTTGCTTCGTCA TGCTGTCTGCCGTTGTTATTT	13748–14522	775
ORF5	GCAACCGTTTTAGCCTGTYTKKT AGGGCATATATCATCACTGGCG		730
ORF7	GCCCCTGCCCACCACG TCGCCCTAATTGAATAGG		650

a Position in the genome of PCR products with respect to the PRRSV/CN/FJGD01/2021 genome.

### Humoral immune response.

Blood samples were collected from all pigs at 0, 4, 7, 11, 14, 17, 21, and 28 dpi to measure specific antibodies against PRRSV N protein using a commercially available enzyme-linked immunosorbent assay (ELISA) kit. One of the five piglets in the PRRSV/CN/FJGD01/2021-infected group became seroconverted at 4 dpi, and all were seroconverted by day 7 postinfection (p.i.). Three of the five piglets in the FJZ03-infected group seroconverted by day 7 p.i., and the remaining two piglets seroconverted by day 11 p.i. Two of the five piglets in the FJ0908-infected group seroconverted by day 11 p.i., and the remaining three piglets seroconverted by ay 14 p.i. All animals were negative for PRRSV-specific antibodies in the control group (Fig. 5D). The above-mentioned data indicated that PRRSV/CN/FJGD01/2021 had higher replication efficiency *in vivo* than the FJZ03 and FJ0908 viruses.

### Bacterial isolation.

Bacteria were isolated from the bronchoalveolar lavage fluid (BALF) of 4 of the 5 piglets inoculated with PRRSV/CN/FJGD01/2021 and from 3 of the 5 piglets inoculated with FJZ03, but from none of the 5 piglets inoculated with FJ0908 and the control group. In the PRRSV/CN/FJGD01/2021-infected group Escherichia coli was detected from three piglets; Staphylococcus and Klebsiella spp. were detected from two piglets. In the FJZ03-infection group, only Escherichia coli and Staphylococcus were detected from two piglets. In contrast, no secondary bacterial infections were detected in the FJ0908-infected group and control group. Streptococcus suis, Haemophilus parasuis, Actinobacillus suis and Mycoplasma hyorhinis were undetected in all piglets throughout the study.

### Macroscopic and microscopic lesions in the lungs.

**FIG 6:**
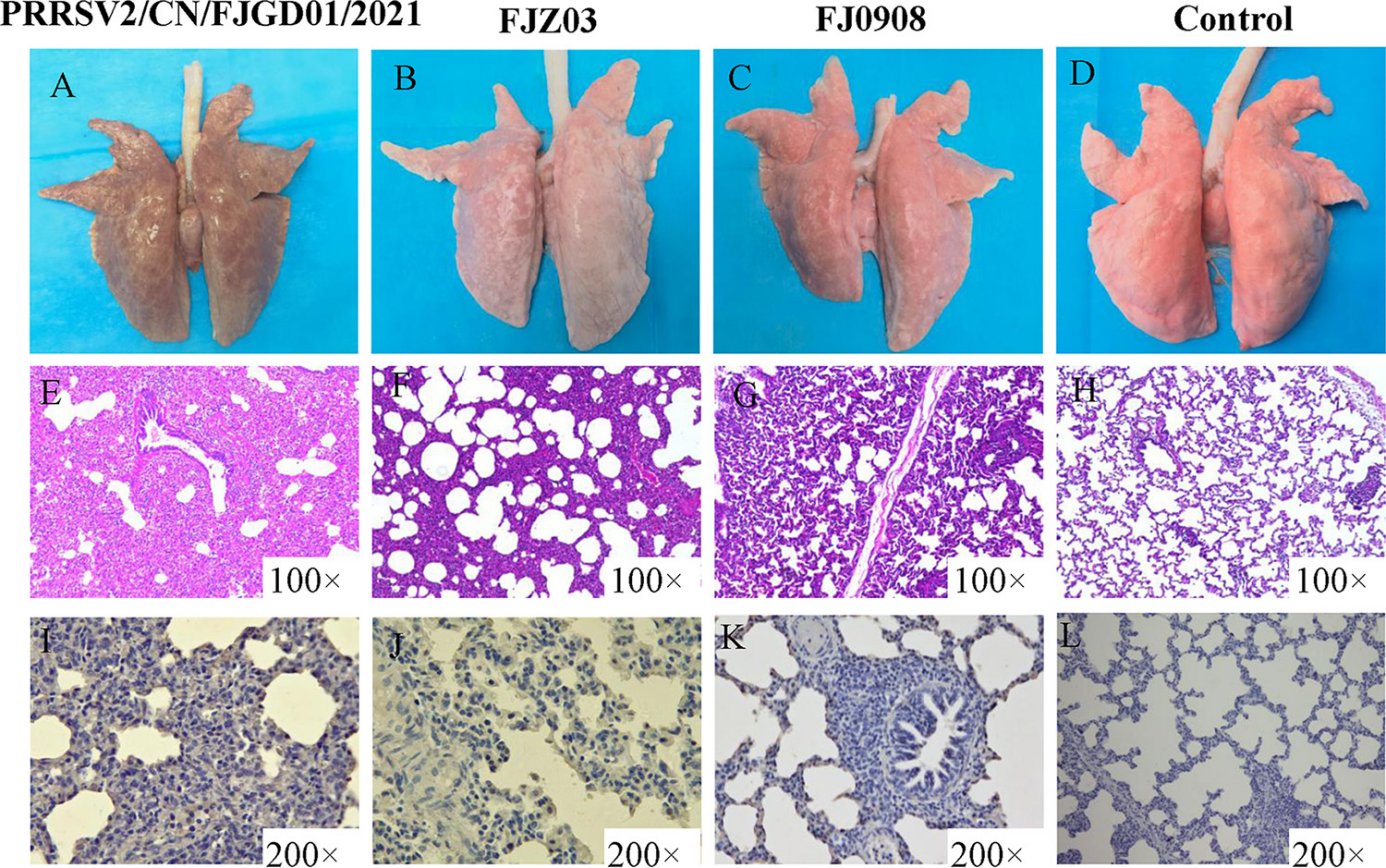
Gross pathology, microscopic lesions and immunohistochemistry examination of the challenged piglets. (A) Moderate tissue consolidation and multifocal, tan mottled indistinct areas was observed in PRRSV/CN/FJGD01/2021 challenged piglets. (B) Mild multifocal lung lesions with scattered hemorrhagic spots were observed in FJZ03-challenged piglets. (C) No obvious gross lung lesions were observed in FJ0908-challenged piglets. (E) Severe interstitial pneumonia with lymphomononuclear cell infiltration and thickening of the alveolar septa of PRRSV/CN/FJGD01/2021-inoculated piglets (magnification, ×100). (F) Moderate interstitial pneumonia with thickened alveolar septa of FJZ03-inoculated piglets (magnification, ×100). (G) Mild interstitial lesions were observed in the FJ0908-inoculated piglets (magnification, ×100). (I to K) PRRSV-positive signals in macrophages and bronchus (magnification, ×200) of lung were detected from piglets inoculated with PRRSV strains PRRSV/CN/FJGD01/2021 (I), FJZ03 (J), and FJ0908 (K). Representative normal lung (D, H, and L) from control piglets.

**FIG 7:**
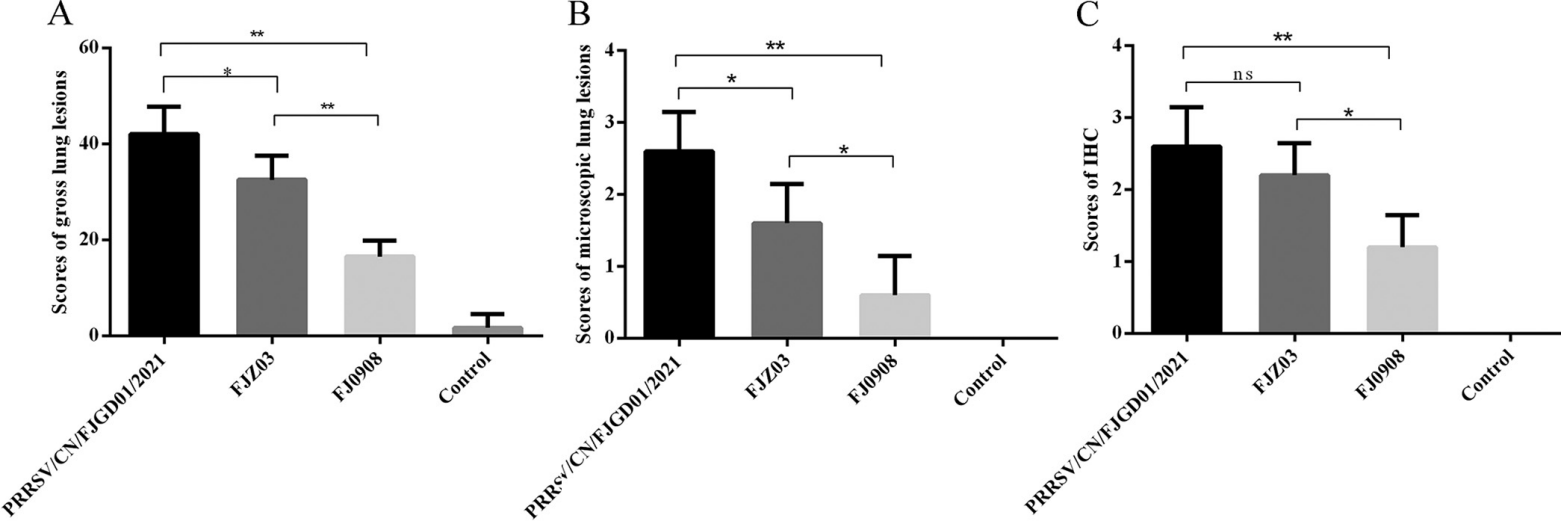
(A to C) Scores of gross lung lesions (A), microscopic lung lesions (B), and IHC (C) of piglets inoculated with three different PRRSV isolates. The significant difference is marked with asterisks (*, *P < *0.05; **, *P < *0.01).

All of the piglets from each group were euthanized and necropsied at 28 dpi. PRRSV/CN/FJGD01/2021-infected piglets demonstrated pneumonia characterized by moderate tissue consolidation and multifocal, tan mottled indistinct areas with scattered hemorrhagic spots (Fig. 6A). FJZ03-infected piglets demonstrated mild multifocal lung lesions (Fig. 6B), whereas FJ0908-infected piglets and control piglets demonstrated no obvious gross lung lesions (Fig. 6C and D). Meanwhile, the mean lung lesion scores of the PRRSV/CN/FJGD01/2021- and FJZ03-infected groups were significantly higher than those of the FJ0908-infected and control groups (*P* < 0.01) (Fig. 7A).

Microscopic lung lesions were similar between PRRSV/CN/FJGD01/2021- and FJZ03-infected groups but varied in severity. Piglets inoculated with the PRRSV/CN/FJGD01/2021 strain showed severe interstitial pneumonia and inflammation characterized by the marked thickening of the alveolar septa, alveolar disappearance and atrophy, and infiltration of neutrophils and lymphocytes (Fig. 6E). The lung sections of the piglets injected with FJZ03 showed moderate to severe interstitial pneumonia with thickened alveolar septa and increased numbers of infiltrating neutrophils and lymphocytes compared to the sections of other piglets (Fig. 6F). However, mild interstitial lesions were observed in the FJ0908-infected group (Fig. 6G), and no obvious microscopic lung lesions were found in the control group (Fig. 6H). The mean microscopic lung lesion scores of piglets in the PRRSV/CN/FJGD01/2021-infected group were significantly higher than those of the piglets in the FJZ03- and FJ0908-infected groups (*P* < 0.05) (Fig. 7B).

PRRSV antigens in the lungs of each group of piglets were examined using immunohistochemistry (IHC). The PRRSV antigen was mainly detected in the macrophages and within the alveolar wall cells in the lung tissue of PRRSV/CN/FJGD01/2021-, FJZ03-, and FJ0908-infected piglets (Fig. 6I to K). No PRRSV-positive antigens were observed in the lungs of negative-control piglets (Fig. 6L). The lung IHC scores of the PRRSV/CN/FJGD01/2021- and FJZ03-infected piglets were significantly higher than those of the FJ0908-infected piglets (*P* < 0.05) (Fig. 7C).

### PRRSV-MLV provides incomplete protection against the heterologous strain PRRSV/CN/FJGD01/2021.

**FIG 8:**
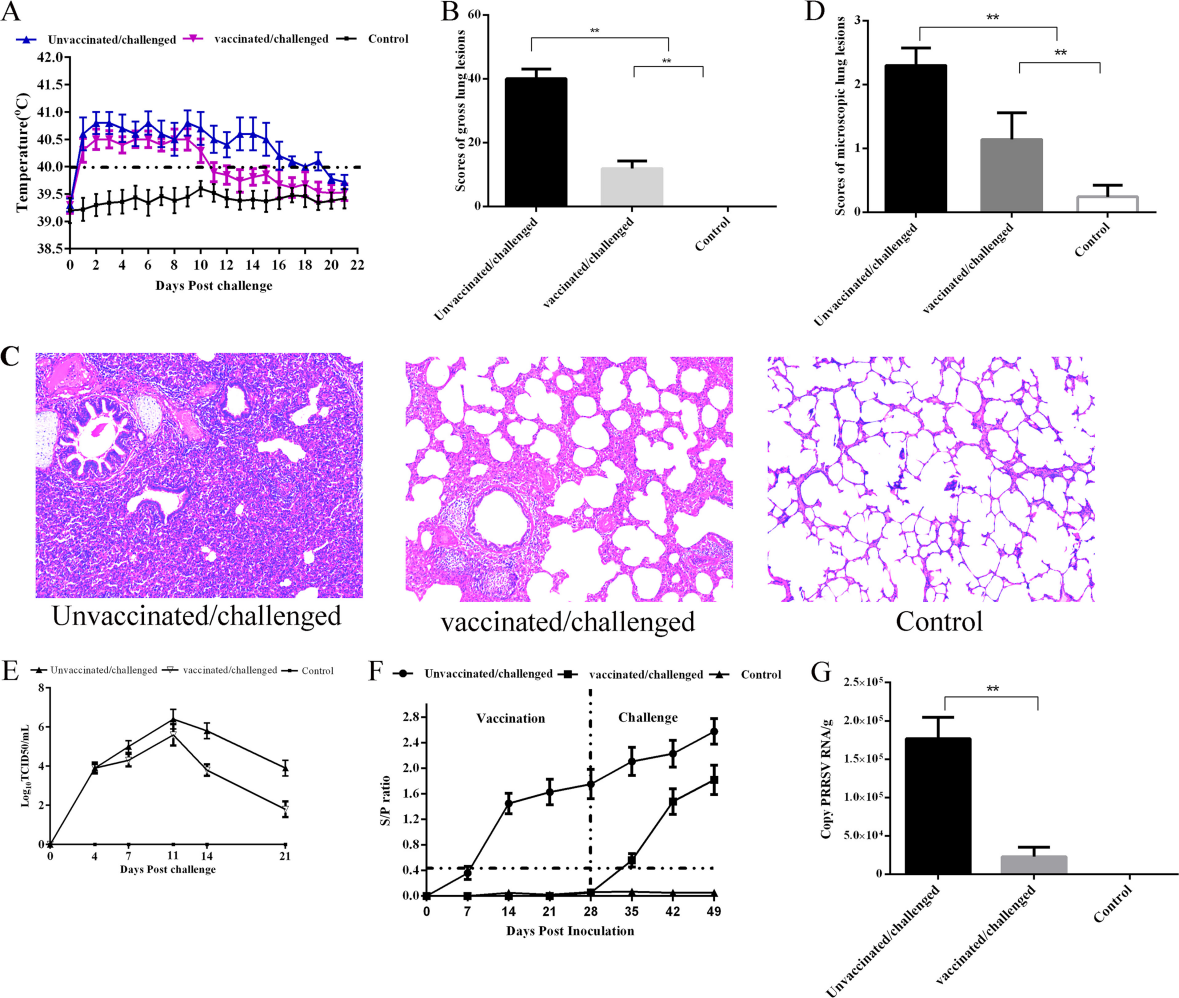
Rectal temperature, gross and microscopic examination, viral loads, antibody titers, and virus RNA load in lungs of piglets. (A) Dynamics of rectal temperature after PRRSV/CN/FJGD01/2021 challenge. Data are expressed as the mean ± SD (error bars) of every group, and the fever cutoff value was set at 40.0°C. (B) Mean scores of lung gross lesions based on the percentage of lung area affected. (C) Lung microscopic pathological lesions (magnification, ×100). (D) Lung microscopic lesion scores based on the severity of interstitial pneumonia. (E) Viral loads in serum after challenge with RRSV/CN/FJGD01/2021. (F) PRRSV-specific antibodies in every group after vaccination and/or PRRSV/CN/FJGD01/2021 challenge. The threshold for seroconversion was set at an S/P ratio of 0.4. Data are expressed as the mean ± SD (error bars) of every group. (G) Virus RNA load in lungs of piglets at 21 dpc (log_10_ copies/g) were measured by real-time PCR. The significant difference is marked with asterisks (*, *P < *0.05; **, *P < *0.01).

The mean rectal temperatures of piglets of the vaccinated group showed fever at 1 day postchallenge (dpc), which lasted for consecutive 10 days, whereas mean rectal temperature was above 40°C during 1 to 19 dpc in the unvaccinated group (Fig. 8A). One piglet in the vaccinated group developed similar PRRSV-specific clinical symptoms, including coughing, dyspnea and tachypnea.

At necropsy, gross lung lesions of piglets characterized by interstitial pneumonia were found in both the vaccinated and unvaccinated groups; no lung gross lesions were observed in control group (data not shown). Compared with the unvaccinated group, the vaccinated group showed lower lung gross lesion scores with a significant difference (*P < *0.01) (Fig. 8B). The microscopic pathological lesions of piglets were also examined; the lesions, including in interstitial pneumonia characterized by thickening of alveolar septa and infiltration of mononuclear cells, were found in piglets in both the vaccinated and unvaccinated challenged groups (Fig. 8C). Compared with the unvaccinated group, the vaccinated group showed microscopic lung gross lesion scores with a significant difference (*P < *0.01) (Fig. 8D).

Virus loads in sera of the challenged piglets were examined. The results showed that the viral loads in the unvaccinated and vaccinated groups of piglets were at similar levels at 4 dpc; however, the level of PRRSV in unvaccinated piglets was significantly higher than that in piglets that had been immunized at 14 dpc and 21 dpc (*P < *0.05) (Fig. 8E). After PRRSV/CN/FJGD01/2021 challenge, antibody levels in all vaccinated group were increased and higher than those of the unvaccinated group (Fig. 8F). Moreover, the virus load in the lungs of vaccinated piglets was significantly lower than that in the unvaccinated piglets at 21 dpc; however, the virus RNA copy number remained high (>log10^4^ copies/g) (Fig. 8G).

## DISCUSSION

PRRS has caused substantial economic losses in the Chinese swine industry since its initial identification in 1996 in China (12). Currently, PRRS has not been controlled well in Chinese swine herds, despite the fact that several imported and domestic commercial modified live virus vaccines are widely used in farms. Previous studies have identified four lineages (lineages 1, 3, 5, and 8) of PRRSV-2 in China, and lineage 1 PRRSVs isolated from China were further classified into sublineage 1.5 (NADC34-like PRRSV) and sublineage 1.8 (NADC30-like PRRSV) (24). More importantly, NADC34-like PRRSV has undergone complex recombination with local Chinese strains (31, 32). In China, the coexistence of different lineages of PRRSV in the field, coupled with the pressure of immunization, may aggravate the evolution of PRRSV, thereby increasing the difficulty of prevention and control of PRRS. In this study, we report a newly emerged PRRSV strain, PRRSV/CN/FJGD01/2021. The complete genome sequence of PRRSV/CN/FJGD01/2021 was determined, and its pathogenicity was evaluated in piglets.

Comparison to PRRSV sequences in the GenBank database indicated that PRRSV/CN/FJGD01/2021 belonged to the NADC30-like PRRSVs. However, PRRSV/CN/FJGD01/2021 shows very low homology (<92%) with NADC30 or NADC30-like PRRSV strains. Moreover, the PRRSV/CN/FJGD01/2021 strain had three discontinuous 131-aa deletion patterns in the Nsp2 gene, which is identical to the NADC30 and NADC30-like strains. All these results indicated that the PRRSV/CN/FJGD01/2021 strain is an NADC30-like virus. However, by further analyzing the viral genomic characteristics in detail, we found that most ORF1 and ORF7 genes of PRRSV/CN/FJGD01/2021 were provided by NADC30-like viruses; in contrast, the major structural protein genes (ORF2a to ORF6) of PRRSV/CN/FJGD01/2021 were provided by NADC34-like viruses. Based on these analyses, we suggest that the PRRSV/CN/FJGD01/2021 isolate is a recombinant virus. Phylogenetic and recombination analyses further confirmed that the PRRSV/CN/FJGD01/2021 strain is a recombinant virus from NADC30-like, NADC34-like, and JXA1-like viruses. Recombination events among PRRSVs have been frequently observed in the field, with the emergence of NADC30-like PRRSVs in China occurring since 2012. Moreover, in recent years, multiple studies have shown that novel PRRSV variants evolved from recombination between two (sublineage 1.8 and lineage 3, sublineages 1.8 and 5.1, or sublineages 1.8 and 8.7) or three lineages (sublineages 1.8, 5.1, and 8.7 or sublineages 1.8, 8.7 and lineage 3) exhibit various degrees of pathogenicity or virulence in pigs (25–28, 30, 33). Recently, a new PRRSV strain, FJLIUY-2017, derived from recombinant strains of four lineage strains (sublineages 1.8, 5.1, and 8.7 and lineage 3) was identified in the field (34). In this study, the PRRSV/CN/FJGD01/2021 strain is also a recombination virus among three sublineages, 1.5, 1.8, and 8.7, of PRRSV-2; to our knowledge, our study is the first to report the recombination pattern between NADC30-like, NADC34-like, and JXA1-like viruses.

Under experimental conditions, the pathogenicity of NADC34-like PRRSV from the United States, Peru, and China in pig has broad variations in virulence (21, 23, 35–37). Piglets inoculated with NADC34 and IN/2014/ISU-5 showed obvious viremia, which caused 2 of 14 piglets to die within 2 weeks, while IA/2014/ISU-2 had little effect on pig growth (21). Similarly, NADC34-like strains isolated in China in piglets exhibited different pathogenicities. The NADC34-like PRRSV strains PRRSV-ZDXYL-China-2018-1 and HLJDZD32-1901 have been demonstrated to have various levels of pathogenicity in piglets. Isolate ZDXYL-China-2018-1 induced persistent fever and moderate dyspnea; in contrast, isolate HLJDZD32-1901 infection caused only mild clinical symptoms in piglets (23, 35). In this study, the NADC34-like strain FJ0908-infected piglets showed very mild symptoms of disease, including no pyrexia, mild gross lung lesions, and little effect on piglet growth. These results are similar to those of HLJDZD32-1901- and IA/2014/ISU-2-infected piglets (21, 35), but different from those of IA/2014/NADC34-infected piglets (21).

Generally, the duration and severity of fever and virulence of PRRSV are closely related to its pathogenic characteristics (38, 39). In this study, piglets inoculated with the PRRSV/CN/FJGD01/2021 strain had a persistently higher fever (>40°C for 2 weeks), obvious clinical signs, and interstitial pneumonia compared to the FJZ03- and FJ0908-infected group throughout the experiment. Previously reported data have revealed that viral loads *in vivo* are one of the measures of virulence and pathogenicity of PRRSV (40). PRRSV/CN/FJGD01/2021-infected piglets had higher viremia than FJZ03 and FJ0908 over the course of infection, suggesting that PRRSV/CN/FJGD01/2021 has higher replication efficiency in piglets. Additionally, PRRSV/CN/FJGD01/2021-infected piglets lost body weight more rapidly than FJZ03- and FJ0908-infected piglets. In combination, our studies indicate that PRRSV/CN/FJGD01/2021 exhibited greater pathogenicity than FJZ03 and FJ0908 but lower pathogenicity than Chinese HP-PRRSVs (16, 28, 41–43).

Clinical symptoms of PRRSV infection in the field are believed to be determined by various factors, including management, immune status, PRRSV virulence, the degree of virus replication in the pig, the age and breed of the animal, and coinfections with other pathogens (21, 44). Previous studies suggested that coinfections of PRRSV with Streptococcus suis and Haemophilus parasuis would increase pig morbidity and mortality, and these deaths may be attributed to secondary bacterial infections (45–48). In this study, we observed a 0% mortality rate in PRRSV/CN/FJGD01/2021-infected piglets, which was much lower than that of the original PRRS outbreak swine herd report. A possible explanation for the difference may be that the use of piglets with a very high sanitary status, appropriate stocking density, and health management in the present experimental study prevented the bacterial infections (such as Streptococcus suis and Haemophilus parasuis) that contribute to more overt mortality (40, 47, 49, 50). These data suggested that severe disease seen in the field is caused by a combination of factors, including PRRSV (51). However, early identification of emerging PRRSV isolates is important for developing effective strategies to prevent and control PRRSV infection. The common feature of NADC34-like PRRSV outbreaks is the large number of sow abortions (21, 22, 35). PRRSV/CN/FJGD01/2021- and FJ0908-infected pigs have a high abortion rate in the field; therefore, it is necessary to further investigate the role of NADC34-like PRRSV in the pathogenesis of pregnant gilts and sows.

Several pathogenicity studies have shown that recombination not only leads to the generation of a novel PRRSV variant, but also plays an important role in PRRSV virulence (27–30, 33, 52). As in the above-mentioned studies, our results support the concept that recombination and virulence are associated. The recombinant virus PRRSV/CN/FJGD01/2021 in this study had higher virulence than the parental virus NADC34-like (FJ0908) and NADC30-like (FJZ03) strains without recombination. Previous studies have shown that HP-PRRSVs, including JXA1, TJ, and JXwn06, are fatal for piglets. These viruses are characterized by high fever, high morbidity (50% to 100%), and high mortality (20% to 100%) (15, 16). A persistent high fever is one of the most important symptoms of HP-PRRSV infection (53). Furthermore, a previous study also suggested that the ORF1a gene of HP-PRRSV plays an important role in viral replication, cytokine response, and lung damage both *in vivo* and *in vitro* based on the reverse genetic system of HuN4 and HuN4-F112 (53). Similar outcomes were described in an experimental study of a virulent PRRSV infectious clone (FL12) by Kwon et al., who pointed out that the Nsp3 to 8 (ORF1a) and ORF5 regions were the main determinants of virulence (54). In addition, previous experimental results showed that Nsp9 and Nsp10 contribute to the fatal virulence of HP-PRRSV for piglets (55). In the current study, the parental virus HP-PRRSV provided a small region (Nsp3 to 5 and Nsp7 to 9) for PRRSV/CN/FJGD01/2021 recombinant virus; in particular, a persistently high fever in the PRRSV/CN/FJGD01/2021 group was similar to the parental HP-PRRSVs, indicating that the genomic components of HP-PRRSVs (Nsp3 to 5 and Nsp7 to 9) may enhance the virulence of the PRRSV/CN/FJGD01/2021 isolate. Surely, the functions of Nsp3 to 5 and Nsp7 to 9 and their influence on pathogenicity require further investigation using a reverse genetics system.

Reduction of PRRSV viremia and virus load in the tissues is an important parameter to evaluate the efficacy of the PRRSV vaccine (56). In this study, Ingelvac PRRS MLV reduced virus titers and shortened the period of fever in vaccinated challenged groups; however, the virus RNA copy number remained high in the lungs at 21 dpc. In addition, pathological analyses revealed that Ingelvac PRRS MLV was partially efficacious at reducing lung gross and microscopic lesions in vaccinated challenged piglets. Together, these data suggest that the commercial PRRSV vaccine provides partial cross-protective efficacy against PRRSV/CN/FJGD01/2021 infection.

In summary, our findings indicate that PRRSV/CN/FJGD01/2021 is a moderately virulent PRRSV, and Ingelvac PRRS MLV vaccine provided incomplete cross-protection against the heterologous PRRSV/CN/FJGD01/2021 strain. Therefore, it is urgent to develop safe and effective vaccines for controlling PRRSV.

## MATERIALS AND METHODS

### Virus isolation.

Serum samples were collected from a pig herd experiencing 20% abortion of sows, and 10% of weaned piglets died in 2021 in Fujian Province, China. Additionally, the piglets were also diagnosed with severe secondary bacterial infections with Streptococcus suis and Haemophilus parasuis. PRRSV infection was confirmed using a real-time reverse transcriptase (RT)-PCR kit (Beijing Anheal Laboratories Co., Ltd.) according to the manufacturer’s instructions. MARC-145^CD163^ cells were used for virus isolation from positive samples as described previously (14, 57, 58). The supernatants of the virus were identified by indirect immunofluorescence assay (IFA) and real-time RT-PCR (55). The isolate was plaque-purified three times, and its third passage on MARC-145^CD163^ cells was subsequently used for the following studies. The NADC30-like strain FJZ03 (isolated in 2013, GenBank accession number KP860909) and NADC34-like strain FJ0908 (isolated in 2018, GenBank accession number MK202794) were used as positive controls (22, 27). Growth properties and kinetics of the viruses PRRSV/CN/FJGD01/2021, FJZ03, and FJ0908 were assessed in porcine alveolar macrophages (PAMs) and MARC-145^CD163^ cells as previously described (57).

### RT-PCR and genomic sequencing.

Viral genomic RNA was extracted from cell culture supernatants of the infected cells using a viral nucleic extraction kit (Tiangen Biotech Co., Ltd., Beijing, China) and used as a template for RT-PCR assays. To obtain the whole-genome sequence of PRRSV, nine pairs of primers (A to I) were designed to amplify the viral genome (Table 3). Additionally, to confirm the full genome of PRRSV, we resequenced the full-length genome of PRRSV from the purified virus and clinical samples using seven other pairs of primers (J to P) as listed in Table 3. Additionally, we also resequenced the flank regions around breakpoints from clinical samples (lung, spleen, lymph node, and serum) and purified virus (Table 3). The PCR products were purified using the Universal DNA purification kit (Tiangen Biotech Co., Ltd.) and cloned into the pEASY-blunt cloning vector (TransGen, China) according to the manufacturer’s instructions. In order to ensure a consensus sequence of each fragment, at least three recombinant clones were sent to the Ruibo Life Technologies Corporation (Beijing, China) for sequencing in both directions by using the Sanger approach. The full-length genomic sequence of PRRSV was assembled using Lasergene software (DNASTAR, Inc., Madison, WI, USA).

### Sequence data analysis.

In total, 39 representative PRRSV-2 genomes, including three HP-PRRSV vaccine strains (JXA1 P80, TJM F-92, and HuN4-F112), and one PRRSV-1 genome available in the GenBank database were used for comparative sequence analyses and molecular evolutionary analyses in the present study. Phylogenetic analysis was constructed from aligned sequences by the neighbor-joining method with MEGA software (version 7.0), and the reliability of the tree was evaluated by bootstrapping using 1,000 replicates according to previous studies (59).

We analyzed recombination events and breakpoints in PRRSV using seven methods (RDP, BootScan, GENECONV, Chimaera, MaxChi, SiScan, and 3Seq) in the recombination detection program 4.10 (RDP 4.10). A recombination event was identified when at least five of the seven methods reported recombination signals in RDP 4.10, with the highest acceptable *P* value being 0.01 (60). Additionally, the potential recombination events estimated by the RDP 4.10 software were further analyzed using SimPlot 3.5.1 software with a 200-bp window width and a 20-bp step size to confirm the potential parental strains (61).

### Animal experiment.

A total of 19 4-week-old piglets were purchased from a commercial pig herd with a PRRSV-, PCV2-, PRV-, and CSFV-free diagnosis. The piglets were blindly divided into four groups, including one control group with four piglets (group 1) and three experimental groups (groups 2 to 4), each with five pigs. Each piglet in groups 2 to 4 was inoculated intranasally (2 mL) with PRRSV/CN/FJGD01/2021 (2 × 10^5^ TCID_50_), FJ0908 (2 × 10^5^ TCID_50_), or FJZ03 (2 × 10^5^ TCID_50_), respectively. Four piglets in the control group were inoculated intranasally with 2 mL of Dulbecco’s modified Eagle’s medium (DMEM). The different groups were housed in separate rooms with separate ventilation.

Rectal temperature was recorded daily from 0 to 28 days postinoculation (dpi). Blood samples were collected at 0, 4, 7, 11, 14, 17, 21, and 28 dpi to detect viremia via an IFA microtitration infectivity assay (38). Nasal swab samples were also collected on the same day as blood samples and used for viremia detection by real-time quantitative PCR (qPCR) as previously described (35, 62). Additionally, animals were weighed at 0, 7, 14, 21, and 28 dpi. All animals were euthanized at 28 dpi for necropsy, and lung tissues were collected and fixed in 10% neutral buffered formalin for histopathology and immunohistochemistry (IHC) with monoclonal antibody 1AC7 for PRRSV N protein (Ingenasa, Spain). A scoring system for lung gross lesion and lung microscopic lesion (from 0 to 4) was applied as previously described (63, 64). The number of positive cells in lung sections was estimated using a ranked score of 0 to 4 according to a previously described method (64): 0 (no positive cells), 1 (1 to 10 positive cells), 2 (11 to 30 positive cells), 3 (31 to 99 positive cells), and 4 (≥100 positive cells). Bacterial culture and bacterial identification of bronchoalveolar lavage fluid (BALF) were performed as previously described (50).

### Virus titration.

All serum samples collected on different days were cultured on MARC-145^CD163^ cells for virus isolation (65). Positive serum samples were subsequently titrated by serial dilution on MARC-145^CD163^ cells to determine viral titers of PRRSV in the unit of 50% tissue culture infective dose per mL (TCID_50_/mL) through IFA as previously described (64).

To quantify the PRRSV load in nasal swabs and lung samples, PCR primers and a probe were designed based on ORF7 as previously described (35). The quantification of the viral load in nasal and lung samples was performed against a standard curve constructed from known amounts of serially diluted ORF7-based plasmids ranging from 10^0^ to 10^9^ copies/μL.

### Serology.

Serum samples obtained at 0, 4, 7, 11, 14, 17, 21, and 28 dpi were analyzed using ELISA with the PRRS virus antibody test kit 2XR (IDEXX Laboratories, Inc., Westbrook, ME, USA) according to the manufacturer’s instructions. Samples were considered positive for antibodies against PRRSV when their sample-to-positive (s/p) was >0.4.

### Efficacy evaluation of a commercial vaccine against PRRSV/CN/FJGD01/2021.

A total of 15 21-day-old healthy piglets that were free of PRRSV, PCV2, PRV, and CSFV were randomly divided into 3 groups (5 piglets in each group). The piglets in group 1 (MLV vaccinated plus PRRSV/CN/FJGD01/2021 challenge group, vaccinated group) were vaccinated intramuscularly with a single dose of Ingelvac PRRS MLV based on the manufacturer’s recommendations on day 0. The piglets in group 2 (unvaccinated plus PRRSV/CN/FJGD01/2021 challenge group, unvaccinated group) and group 3 (unvaccinated unchallenged, control) were mock vaccinated with phosphate-buffered saline (PBS) on the same day. Each piglet in group 1 and group 2 was challenged with PRRSV/CN/FJGD01/2021 (2 × 10^5^ TCID_50_/pig, 2 mL) at 28 days postvaccination (dpv) (0 days postchallenge, dpc). Each piglet in the control group was inoculated with 2 mL of DMEM.

Rectal temperature was recorded daily. Serum samples collected at 0, 7, 14, 21, and 28 dpv and 4, 7, 11, 14, and 21 dpc were tested for antibodies specific for N protein of PRRSV using a commercially available ELISA kit (IDEXX Laboratories, Inc., Westbrook, ME, USA) and viremia via IFA as mentioned before. All animals were euthanized at 21 dpc for necropsy; all the samples were treated as mentioned before.

### Data analysis.

Data are expressed as the mean ± standard deviation (SD). Determination of statistical differences among groups in terms of body temperature, weight gain, and viral load, as well as histopathological and immunohistochemistry scores of lungs was performed using a one- or two-way analysis of variance (ANOVA) with GraphPad Prism software (version 6.0). A *P* value <0.05 was considered statistically significant.

### Ethical approval.

In this study, piglets’ clinical samples used had obtained a written consent from farm owners. The piglet clinical samples used in this study were collected in strict accordance with the Animal Ethics Procedures and Guidelines of the People’s Republic of China. The procedures of animal handling and experimentation performed in this study were approved by the Longyan University animal ethics committee (permit no. LY20210010X).

### Data availability.

The data used to support the results of this study are included within the article and in the GenBank database.
